# Oxidative Stress-Guided Gold Nanoparticles for Cancer Theranostics

**DOI:** 10.3390/antiox15050641

**Published:** 2026-05-18

**Authors:** Yubin Jin, Jiaxuan Zhu, Yang Yang, Zhuhu Li, Yunzhi Qin

**Affiliations:** 1 The Affiliated Hospital of Yanbian University (Yanbian Hospital), Yanji 133000, China; 1225111496@ybu.edu.cn (Y.J.); yangyang@ybu.edu.cn (Y.Y.); 2 Key Laboratory of Pathobiology, Yanbian University, State Ethnic Affairs Commission, Yanji 133000, China; 1225111507@ybu.edu.cn

**Keywords:** gold nanoparticles, oxidative stress, reactive oxygen species, cancer theranostics, radiosensitization, photothermal therapy, photodynamic therapy, tumor microenvironment

## Abstract

Gold nanoparticles offer a versatile platform for cancer theranostics because their high atomic number can enhance X-ray energy deposition, their plasmonic properties support photothermal and photoacoustic applications, and their surfaces allow drug loading and molecular targeting. However, therapeutic benefit remains heterogeneous because tumor uptake, intratumoral coverage, and subcellular localization determine whether deposited gold can be converted into biologically effective damage. Redox context further shapes this conversion by determining whether AuNP-triggered physical or catalytic events can overcome local buffering and propagate into durable injury. During radiotherapy, AuNPs increase local secondary electron release and ROS formation, which can intensify DNA damage when GSH-dependent peroxide detoxification, thioredoxin-related buffering, and KEAP1-NRF2-regulated antioxidant responses are insufficient to contain the redox burden. In catalytic systems, Au-containing nanozymes can convert endogenous H_2_O_2_ into highly reactive radicals and may simultaneously deplete glutathione, thereby amplifying mitochondrial dysfunction and lipid peroxidation. During photoactivation, plasmonic heating and photosensitizer coupling further reshape ROS generation in a time-dependent and location-dependent manner. On the diagnostic side, CT or spectral CT can quantify tumor gold burden and coverage, whereas ROS-responsive photoacoustic, SERS, or fluorescence probes can report treatment-related oxidants and verify whether redox activation has occurred within the tumor. Clinical translation will therefore depend on quantification-guided dosing, definition of spatial coverage and activation timing, standardized redox-response readouts, and long-term safety evaluation.

## 1. Introduction

Use of radiotherapy has largely been restricted due to the limited therapeutic window between tumor response and normal tissue toxicity [1,2]. Increasing the radiation dose improves the therapeutic efficacy against the tumor. However, this is often associated with dose-limiting toxicity to surrounding normal tissues. In addition, if there is tumor recurrence after treatment, or if the tumor responds initially to treatment but develops resistance to further radiation, dose escalation can be difficult [3]. Most anti-cancer agents work via induction of oxidative damage to cancer cells. Radiotherapy also works via this mechanism to cause cancer cell death. However, due to upregulation of reactive oxygen species (ROS) scavengers, as well as activation of cytoprotective stress response programs, especially in hypoxic regions of the tumors, cancer cells could develop tolerance toward oxidative insult and thus survive despite being exposed to cytotoxic conditions.

AuNPs have attracted sustained interest in oncology because they can localize energy deposition, generate trackable signals, and support multifunctional surface engineering. However, their clinical performance remains variable because particle delivery, intratumoral microdistribution, and intracellular localization differ markedly across tumors [4,5]. As a result, physical activation does not necessarily translate into effective biological damage. Because many AuNP-enabled therapeutic modules ultimately converge on ROS generation or redox disruption, tumor antioxidant defenses, oxygenation status, and subcellular localization are likely to influence whether deposited gold can be translated into effective tumor cell damage [5,6].

Oxidative stress is central to AuNP theranostics not as an isolated background condition, but as the biological interface through which nanoparticle localization and activation are converted into injury or remain limited to reversible perturbation. In AuNP-enabled radiotherapy, catalytic therapy, and photoactivated therapy, deposited gold initiates different physicochemical events, including secondary electron release, plasmonic heating, photosensitizer-coupled ROS production, or catalytic H_2_O_2_ conversion. Whether these events become therapeutically meaningful depends on lesion-specific oxygenation, H_2_O_2_ availability, antioxidant buffering capacity, and subcellular vulnerability [3,7,8]. These variables shape redox permissiveness and thereby influence whether AuNP activation culminates in DNA damage, lipid peroxidation, mitochondrial dysfunction, or only limited and reversible perturbation.

**Figure 1:**
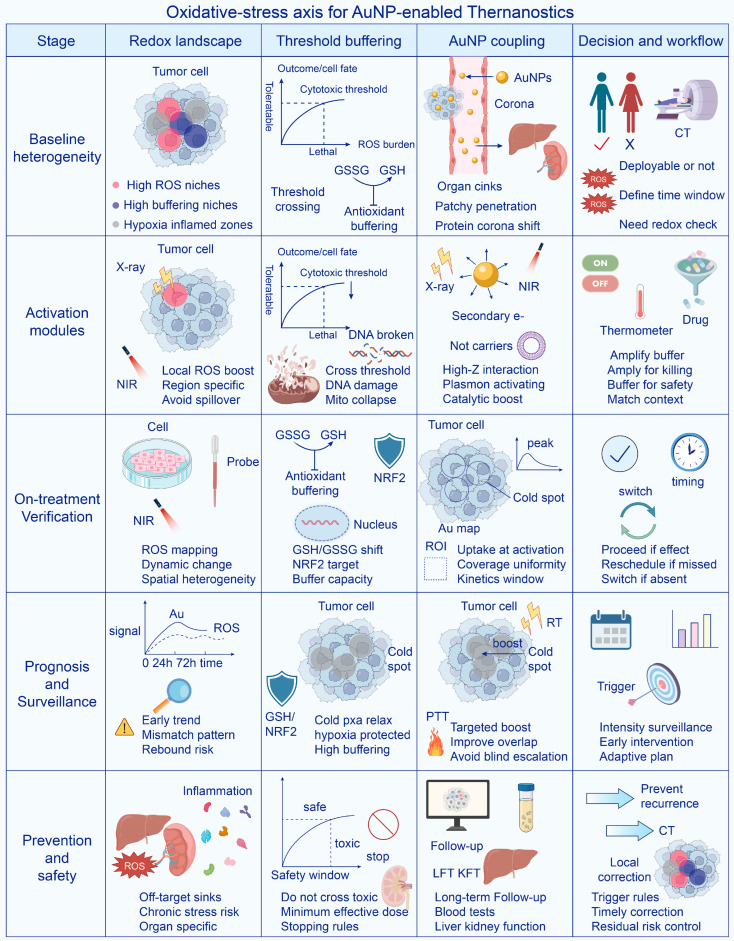
AuNP-centered oxidative-stress framework in cancer. This figure summarizes how AuNPs integrate delivery behavior with oxidative-stress regulation across cancer diagnosis, therapy, prognosis, and prevention. Diagnostic applications define tumor accessibility and redox heterogeneity, while therapeutic strategies exploit tumor-selective oxidative amplification or redox disruption to improve efficacy. Prognostic monitoring and preventive surveillance further extend this framework by tracking delivery, response, and recurrence-related redox changes.

This framework also extends to diagnosis, treatment evaluation, and follow-up. Quantitative AuNP delivery metrics define whether activation is feasible, whereas redox-associated readouts become informative only when they are linked to AuNP distribution, activation timing, and downstream damage endpoints [9,10,11]. Under this view, oxidative-stress-guided AuNPs are best understood as systems in which redox biology is integrated into lesion qualification, activation scheduling, response verification, and safety assessment, thereby connecting nanoparticle delivery with clinically meaningful theranostic decision making [10] (Figure 1).

To explore these concepts further, we conducted a comprehensive literature search using PubMed and Web of Science for English-language papers published between January 2021 and January 2026. Our search focused on keyword combinations related to AuNPs, oxidative stress, reactive oxygen species, tumor diagnosis, imaging, therapy, radiosensitization, photothermal and photodynamic modalities, prognosis, and prevention. In addition to database searches, we complemented our review with citation tracking from key primary studies. We excluded studies not focused on cancer, reports not involving gold-based nanosystems, and articles lacking relevance to the diagnostic, therapeutic, prognostic, or preventive aims of this review. We prioritized studies that established AuNP-linked oxidative stress mechanisms and reported quantifiable redox or ROS-related endpoints, incorporating human evidence where available, alongside data from animal and cellular models.

## 2. Coupling of ROS Sources, Antioxidant Networks, and Threshold Effects in the Tumor Microenvironment

Tumor-associated oxidative stress may not be just a static state of increased ROS levels. Rather, it represents a dynamic equilibrium established by the degree of ROS production, the capacity of detoxification, and spatiotemporal compartmentalization, which altogether determine immunosuppression, vascular abnormalities, stromal barriers, and therapeutic responses [12]. ROS production and signaling are largely compartmentalized, with defined microdomains forming within mitochondria, endoplasmic reticulum, peroxisomes, and the nucleus/cytosol [13]. In terms of mitochondria, the main intracellular source of ROS production derives from the electron transport chain and is mainly associated with complexes I and III; at least some proportion of ROS formation will be localized near the site of scavenging systems that can neutralize them [14]. Moreover, oncogenic signaling can increase anaplerotic inputs and NADH supply, which drives electron flux through the respiratory chain and enhances mitochondrial ROS production. Thus, ROS is coupled to tumor metabolism. In addition to mitochondria, members of the nicotinamide adenine dinucleotide phosphate (NADPH) oxidase (NOX) family represent another important enzymatic source of ROS, including NOX1-5 and dual oxidase 1 and 2 (DUOX1/2), which mainly produce O_2_•^−^ and H_2_O_2_. Under hypoxic microenvironmental conditions, HIF-1-dependent metabolic and mitochondrial regulation can modulate ROS generation and accumulation, potentially sustaining high ROS levels that support tumor progression [13]. This regional variability helps explain why oxidative stress does not translate into a uniform therapeutic background across the tumor.

Antioxidants act as buffers against ROS stress. Glutathione is the most important component of this network since it contributes both to direct scavenging of ROS and cofactor production for peroxide reduction. The glutathione cycle starts with synthesis catalyzed successively by GCLC and GCLM (also called GCL) and then by GSS/GS. This pathway provides reduced glutathione (GSH) which is used for peroxide reduction by glutathione peroxidases (GPXs), GPX-like PRDX6, and lipid peroxide clearance by glutathione S transferases (GSTs) [14]. Regeneration of GSH from its oxidized form (GSSG) is achieved by glutathione reductase using NADPH as the electron donor. NADPH is mainly produced by glycolysis and pentose phosphate pathway [15]. Finally, conversion of ROS can be performed, for instance, by the dismutation of O_2_•^−^ by superoxide dismutase (SOD) family members or H_2_O_2_ decomposition into H_2_O and O_2_ by catalase located in peroxisomes [14]. The net redox state of tumor cells therefore reflects buffering capacity as much as oxidant generation.

One of the main transcriptional programs designed to reduce ROS levels is the KEAP1-NRF2-ARE pathway, which responds strongly even to mild oxidative stress [1]. The main targets of this pathway are HO-1, NQO1, GCLC, GCLM, TXN1, PRDX1, SRXN1, SLC7A11, GSR, and G6PD, which help maintain glutathione (GSH) synthesis and turnover, cysteine/cysteine influx, and thioredoxin (Trx)-based defense. In addition to NRF2, the Trx system has also been found to have a role in redox signaling [9].

However, ROS-associated effects become therapeutically meaningful only when oxidative stress is shifted beyond the tolerable range of tumor cells while remaining within the safety range of normal tissues. Threshold refers to the biological boundary at which oxidative stress in tumor cells ceases to be primarily adaptive and begins to produce durable downstream injury, including mitochondrial dysfunction, lipid peroxidation, DNA damage, apoptosis, and loss of clonogenic survival, while normal tissues remain unaffected or only minimally harmed. The therapeutic value of AuNP-enabled redox modulation therefore depends on whether intervention can selectively push tumor cells across this damage-associated boundary without driving normal tissues beyond their tolerable limit. For example, H_2_O_2_ acts as a signaling molecule at lower concentrations, whereas it becomes toxic at higher concentrations [16]. At the molecular level, either pro-apoptotic or cytoprotective mechanisms can be activated depending on whether O_2_•^−^ predominates over H_2_O_2_ [13]. O_2_•^−^, because of its poor diffusibility and low membrane permeability, reacts with NO to generate peroxynitrite (ONOO^−^) as a source of local damage to proteins, lipids, and nucleic acids [1]. Thus, when ROS production exceeds the capacity of cellular antioxidant systems, different transcriptional and damage-response programs are engaged in a context-dependent manner. In therapeutic terms, the critical issue is not the existence of a universal ROS value, but whether oxidative stress remains within a tolerable range in normal tissues while being pushed beyond a damage-associated threshold in tumor cells. If this balance is not achieved, attempts to increase ROS may fail to improve tumor killing and may instead increase unwanted effects such as immunosuppression or toxicity [14]. Because ROS generation, antioxidant buffering, and damage thresholds vary across tumor regions, identical AuNP formulations can produce divergent outcomes unless particle delivery is aligned with local redox vulnerability.

Within the tumor microenvironment, cancer-associated fibroblasts (CAFs), myeloid-derived suppressor cells (MDSCs), tumor-associated macrophages (TAMs), and regulatory T (Treg) cells are key nodes for ROS production and ROS responsiveness. They act as major effectors of an immunosuppressive niche. Pathological expansion of MDSCs can activate JAK/STAT3 signaling and NOX enzymes, thereby increasing ROS production. MDSC-derived ROS is linked to T cell immunosuppression, metastasis, and tumor progression. Inhibiting ROS generation can attenuate immunosuppression and restore CD8^+^ T cell proliferation. TAMs can release H_2_O_2_ and suppress T cell activation. In ROS-rich settings, upregulation of antioxidant responses such as NRF2 and HO-1 is associated with polarization tendencies [14]. Treg suppressive function is shaped by ROS-related transcriptional regulation. Secretion of antioxidant factors such as Trx can increase resistance to ROS-induced apoptosis. Under excessively high ROS, Treg apoptosis may also occur and influence immunotherapy outcomes. On the vascular side, ROS induces VEGF and promotes tumor angiogenesis. NOX-derived ROS and the HIF-1α VEGF axis support an aberrant vascular niche. Antioxidant lowering of ROS may also promote angiogenesis via BACH1 [14]. On the stromal side, CAFs remodel the ECM and promote tumor growth, angiogenesis, invasion, metastasis, and therapeutic resistance. These pro-tumor effects are closely coupled to ROS regulation [16]. Elevated ROS can enhance HIF-1α accumulation and promote myofibroblast-like differentiation and metastatic traits through pathways such as CXCL12 CXCR4. Tumor-cell-derived ROS can also enhance CAF autophagy and provide metabolic support. It can promote CAF mitophagy with mtDNA release and mtDNA uptake by tumor cells can support survival and metastasis. In resistance phenotypes, CAF-derived exosomes can reduce lipid ROS accumulation in tumor cells and promote chemoresistance. More broadly, tumor cells can counter high ROS by increasing antioxidant synthesis or upregulating antioxidant enzymes, thereby acquiring tolerance to ROS-based therapies [14,17].

The major reactive species include O_2_•^−^, H_2_O_2_, •OH, and ONOO^−^. Nitric oxide (NO) can react with O_2_•^−^ and generate ONOO^−^ [1,18]. Excess ROS damage readouts include lipid peroxidation, which can be captured by lipid ROS accumulation [18]. Oxidative DNA damage can be captured by 8-OH-dG and 8-oxoG and can be accompanied by AP sites and single- or double-strand breaks. ROS-mediated inhibition of OGG1 can impair initiation of 8-oxoG repair [13]. Whether these lesions accumulate and translate into stable phenotypes depends on reductive buffering capacity. The GSH/GSSG ratio and GSR- and NADPH-dependent regeneration of GSH from GSSG, supported by PPP-derived NADPH, describe intracellular redox homeostasis. For AuNP theranostics, these redox networks define the local conditions under which deposited gold becomes biologically consequential. Regions enriched in H_2_O_2_ but weakly supported by GSH-GPX detoxification, thioredoxin-peroxiredoxin buffering, or NADPH-dependent reductive capacity are more permissive to oxidative amplification. In contrast, hypoxic, NRF2-high, GSH-rich, or poorly exposed regions may attenuate injury despite measurable Au accumulation. Redox heterogeneity therefore explains why similar levels of tumor-associated gold can yield markedly different biological outcomes. In practical terms, oxidative stress should be interpreted as a spatially heterogeneous conversion condition that interacts with AuNP coverage, retention, intracellular localization, and activation modality to determine whether local initiation becomes durable damage [19].

## 3. Interactions Between Oxidative Stress and AuNPs

### 3.1. From Administration Route and Engineering Variables to In Vivo Exposure and Activation Timing

The clinical translation of AuNPs for cancer applications depends not only on particle engineering but also on how the particles are delivered in vivo. The route of administration is an upstream determinant because it defines the pharmacokinetic setting in which AuNPs circulate, localize, and are cleared. Intravenous delivery relies on sufficient blood persistence and successful vascular extravasation, but it is also constrained by mononuclear phagocyte system uptake and heterogeneous tumor penetration. Intratumoral delivery can increase local Au burden by bypassing the circulation and extravasation bottleneck, although its benefit may remain spatially restricted by incomplete intratumoral spread or leakage from the injection site. Oral delivery is even more formulation-dependent because gastrointestinal degradation, epithelial transport, and first-pass redistribution can markedly restrict systemic availability. Intraperitoneal delivery is most relevant to peritoneal disease, where locoregional exposure may exceed that achieved by systemic dosing, although peritoneal clearance and uneven coverage of tumor nodules remain important limitations. From a translational perspective, the key variables are therefore not only tumor uptake, coverage, and retention kinetics, but also whether the selected administration route can realistically support these requirements under clinically feasible conditions (Table 1).

In this sense, the design-to-benefit cascade begins with whether sufficient in vivo AuNP exposure can be achieved at acceptable dose levels [20,21], then whether intratumoral distribution places the particles within the regions relevant for imaging or irradiation [22], and finally whether enough particles remain at the intended intervention timepoint to support activation and monitoring [23,24]. Each step narrows the conditions under which delivered gold can still be translated into usable therapeutic or diagnostic output.

**Figure 2:**
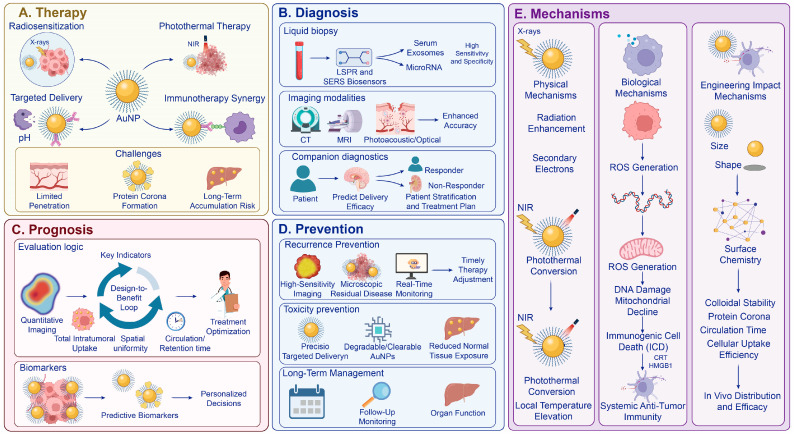
Route-aware engineering-to-biodistribution cascade that defines AuNP deployability and the activation time window. Scheme shape, surface chemistry, and stability affect circulation, clearance, organ uptake, etc., through influencing the formation of protein corona. This further determines tumor uptake and retention, and also defines a period of time window for image-directed therapy induced by radiation or light. This also explains why biodistribution verification and safety monitoring should be carried out before evaluating the therapeutic effect.

Once the administration route has been established, the factors influencing biodistribution, such as particle size, shape and surface chemistry, should no longer be regarded exclusively as determinants. They are also determinants of activation efficiency after tumor arrival. Size influences surface atom fraction, local curvature, and the balance between interfacial reactivity and shielding, thereby affecting how effectively deposited gold can support radiochemical amplification under irradiation [25]. Shape controls aspect ratio, edge sharpness, and plasmon mode distribution, which in turn shifts resonance position, redistributes the near field, and changes the balance between absorption and scattering during optical activation [26]. Surface chemistry then determines whether these core properties remain functionally available in biological media, because coatings regulate colloidal stability, protein corona formation, membrane interaction, intracellular trafficking, and the extent to which the gold surface remains exposed to the surrounding chemical environment [23,24,27]. As a result, particles that reach the tumor at similar bulk levels may still differ markedly in radiosensitizing or photothermal output, because the decisive issue is not only how much gold is delivered, but how efficiently deposited gold couples to X-ray or optical activation after arrival. Colloidal stability under physiological conditions is a basic requirement since aggregation would lead to faster clearance, lower signal reproducibility, and higher levels of nonspecific organ deposition, which poses a safety risk. Polymer-based surface strategies such as polyethylene glycol modification may improve stability and prolong circulation depending on the formulation [24]. Imaging and therapy must therefore be performed at a time when tumor uptake is sufficient to provide both imaging contrast and therapeutic efficacy, but before most of the activity has been cleared from the body or redistributed [24,27]. For cancer theranostics, the central objective is not tumor accumulation alone, but activation-ready exposure, meaning that sufficient gold must be present at the right time, distributed across the relevant tumor regions, and positioned in cellular compartments where physical or catalytic activation can be translated into durable injury (Table 2) (Figure 2).

Importantly, these delivery variables should not be treated as universally linear surrogates of therapeutic efficacy. Current evidence indicates that total tumor accumulation alone is often insufficient to predict treatment benefit, whereas biologically available exposure, spatial coverage of the target volume, intracellular access, and alignment between retention kinetics and activation timing are more closely associated with downstream response [8,32]. In a quantitative imaging study, intracellular nanoparticle exposure predicted nanotherapeutic efficacy more accurately than overall nanoparticle accumulation in tumor mass [8]. In radiosensitization models, matched nanoparticle metal mass uptake correlated with radioenhancement across 2D, 3D, and in vivo systems, with 3D models more faithfully reflecting in vivo behavior [32]. Clinically, nanomedicines have often improved therapeutic index by modifying pharmacokinetics and biodistribution, but lesion-level efficacy thresholds remain context-dependent rather than universal [33]. Accordingly, uptake, coverage, and retention kinetics should be interpreted as upstream exposure variables rather than efficacy surrogates, whereas residual DNA damage, clonogenic loss, apoptosis, and tumor growth control represent downstream response variables that determine whether biologically effective injury has actually been achieved. The final outcome depends on whether activation-ready exposure coincides with a redox-permissive compartment. In this sequence, AuNP localization defines where and when the initiating event can occur, the activation modality defines the type and spatial range of ROS or energy release, and local antioxidant networks determine whether this initiation is buffered or propagated into DNA damage, lipid peroxidation, mitochondrial dysfunction, apoptosis, and clonogenic loss.

From a measurement perspective, AuNPs act as signal-amplifying interfaces that convert local physicochemical events into detectable outputs. The plasmon-driven near-field enhancement supports SERS-based detection by amplifying the Raman signature of reporters located near the electromagnetic hot spot, so as to achieve multi-channel when the tag is designed for stability [34,35]. In order to support the in vivo decision-making process, quantitative readings need to control the external variability, such as excitation conditions and background; ratiometric SERS strategy improves the reproducibility of normalization of the signal of interest to the internal reference peak, and multimodal co-registration reduces the probability of false-positives through anchoring the signal to the anatomical context [36]. Standardization is closely linked with tag uniformity and protective measures, which could reduce reporter loss and environmental interference; preparation conditions should be reported since they determine the batch-to-batch comparability [37].

Under optical irradiation, AuNPs act as plasmonic transducers rather than generic physical amplifiers. Localized surface plasmon resonance arises when incident light drives collective oscillation of conduction-band electrons at the nanoparticle surface, producing a confined electromagnetic field together with wavelength-dependent absorption and scattering [38]. Because this resonance is set by particle size, aspect ratio, curvature, interparticle spacing, and the dielectric properties of the surrounding medium, increasing the diameter of spherical AuNPs generally red-shifts the plasmon band, whereas in gold nanorods a higher aspect ratio shifts the longitudinal mode toward longer wavelengths and can move it into the near-infrared window [38,39,40]. Surface coatings can further shift or damp the resonance by changing the local dielectric environment at the metal interface, so even particles with similar tumor accumulation may differ in optical coupling and photothermal output after arrival [41,42]. For photothermal applications, the decisive variable is not nominal laser power alone but how much of the incident photon flux is actually extinguished and routed into non-radiative decay within the particle. Accordingly, absorption-dominant structures with favorable photothermal conversion efficiency generate heat more effectively than particles whose optical response is weighted toward scattering, even when both are irradiated at the same wavelength and power density [22,43].

Under X-ray irradiation, the high atomic number of gold increases the probability of photoelectric interactions and secondary electron emission in the immediate periparticle volume, so dose amplification is intrinsically nanoscale rather than uniform across the tissue [44,45,46]. This physical gain is therefore constrained by beam energy, by how densely particles populate the irradiated region, and by whether their microdistribution places electron tracks close enough to biologically vulnerable targets to convert local energy deposition into durable damage [47,48]. This conversion is further modulated by nanoparticle design. Smaller AuNPs expose a larger fraction of surface atoms and therefore can support stronger interfacial chemical amplification, whereas surface coatings may preserve intracellular availability but can also modify low-energy electron escape and local radiochemical conditions [49]. Recent work has shown that ROS production and biological radiosensitization vary with both particle size and coating, with smaller AuNPs and selected PEG-capped formulations producing stronger clonogenic radiosensitizing effects in some experimental settings [50]. These observations indicate that surface chemistry is not merely a pharmacokinetic accessory in radiotherapy design. It is part of the mechanism that determines whether nanoscale physical initiation can be propagated into durable chemical and biological injury.

Therefore, the limitation of macroscopic effect is often not determined by basic physics principles, but whether enough particle density can be achieved and whether there is a proper degree of spatial overlap inside the tumor volume [44,47]. The sub-cellular localization also poses another constraint as most internalized particles are trapped inside vesicular compartments, so their closeness to sensitive targets cannot be assured. This can limit the efficient transition from physical initiation to downstream endpoints [48]. Accordingly, biological validation must show not only particle presence but also conversion of local physical amplification into durable damage-related endpoints. Phosphorylated histone H2AX (γ-H2AX) and p53-binding protein 1 (53BP1) foci are the most commonly used direct DNA damage markers for biological validation; they provide early readouts, including residual foci at later time points which indicate delayed repair; clonogenic survival remains the ultimate endpoint most directly linked to treatment efficacy, and sensitization performance can be summarized with enhancement ratio metrics derived from clonogenic assays [50].

In addition, translational feasibility must also consider the balance between efficacy and safety. The biodistribution needs to be tested by both in vivo imaging and ex vivo tissue examination in order to verify whether there is enough accumulation in tumors while minimizing the uptake of surrounding normal organs [51]. For toxicity assessments, both systemic blood analysis and organ-level evaluation should be included. Because gold-based materials are not readily degraded in the body, long-term follow-up is required for clinically intended systems [52].

### 3.2. Pro-Oxidative Amplification and Antioxidative Buffering

AuNP-mediated ROS regulation should be interpreted as a context-dependent conversion process rather than a uniform pro-oxidant or antioxidant effect. In tumor settings, AuNPs can amplify ROS accumulation when particle localization and the activation modality place ROS generation near vulnerable compartments, leading to mitochondrial dysfunction and radiation-associated injury [53]. In other stress settings, AuNPs may instead support redox recovery through NRF2-linked defensive programs and restoration of organelle homeostasis, thereby returning ROS levels to a tolerable range [54]. This bidirectional behavior indicates that the biological effect of AuNPs is governed by the initial redox state, substrate availability, intracellular localization, and antioxidant network capacity. Under pro-oxidant therapeutic conditions, ROS elevation becomes meaningful only when local buffering is exceeded and the signal propagates into mitochondrial dysfunction, membrane damage, DNA injury, and loss of viability. The key translational question is therefore not whether AuNPs increase or decrease ROS in general, but whether their localization and activation generate a compartment-specific redox shift that crosses the local damage threshold and produces measurable biological injury [10].

Prooxidant design makes AuNPs platforms that shift the balance of ROS production and ROS reduction toward greater imbalance. For the PoPD@Au system, the central chain of this system is the peroxidase-like reaction that transforms hydrogen peroxide into highly active radical species. Hydroxyl radical production has been verified by EPR with spin-trapping, and substrate oxidation assays have also confirmed the oxidation effect of this system. To provide sufficient substrates for the oxidation reaction, it possesses a glucose oxidase-like activity which generates extra hydrogen peroxide from glucose; thus, the upstream reaction could be sustained and further amplified. Accordingly, there were higher levels of intracellular total ROS detected at high glucose condition, along with lower cell viability observed [55]. In addition, GSH exhaustion decreased the cellular redox-buffering capability and limited the ROS scavenging effect, so that the pro-oxidant effect would last longer. Mitochondrion was involved in the downstream event, and the JC-1 mitochondrial membrane potential probe revealed a loss of membrane potential, which agreed with live/dead staining and increased cell death. And they were translated into tumor suppression in vivo [56]. Taken together, these findings indicate that sustained oxidative injury is most likely to emerge when substrate generation, ROS conversion, and buffering collapse occur within the same tumor compartment, allowing redox perturbation to extend beyond probe-detectable fluctuation and develop into irreversible cellular damage. Another example of pro-oxidant mechanism could be found in the RGD-AuNPs-SAHA system. More intracellular total ROS accumulation and apoptosis were observed in both A549 and patient-derived tumor organoid models, which indicated a relationship between pro-oxidant response and cytotoxicity [53].

The coupling between radiosensitization and oxidative stress can be summarized in two forms. The first links a physical initiation to a DNA-damage endpoint. Enhanced X-ray absorption by AuNPs is used as the starting point for sensitization. At the outcome level, increased γ-H2AX-positive cells in tumor tissues indicate accumulated DNA damage, while elevated terminal deoxynucleotidyl transferase dUTP nick end labeling (TUNEL) signals support enhanced apoptosis, together with tumor growth inhibition as convergent evidence for synergy [53,57]. At the second level, the tumor redox background determines whether this local amplification is sustained or blunted, particularly under hypoxic conditions that weaken oxygen-radical-mediated injury. Because hypoxia can weaken the oxygen-free-radical effects of radiotherapy, suppression of hypoxia-inducible factor 1α (HIF-1α) and downregulation of vascular endothelial growth factor (VEGF) are used to explain improved radiosensitivity, and inhibition of Janus kinase 2 (JAK2) and signal transducer and activator of transcription 3 (STAT3) signaling that converges on VEGF is consistent with a downshift of the hypoxia-vascular program.

Evidence from non-tumor stress models shows that AuNP exposure can also engage antioxidant restoration and organelle recovery under specific biological conditions [54,58,59,60]. In the present context, this observation is useful mainly because it highlights the strong dependence of AuNP effects on baseline redox state, intracellular context, and damage susceptibility, all of which influence whether ROS modulation remains adaptive or progresses toward therapeutically relevant injury.

## 4. Diagnosis

Diagnosis is not merely a matter of biomarker detection. Its primary role is to determine whether an AuNP-enabled intervention is deployable, to define the optimal activation window, and to identify redox-relevant heterogeneity that may limit therapeutic benefit [61]. Quantitative reporting will likely need to take account of within chip as well as between batch variability metrics because there could be drifts in the signal-to-concentration relationship leading to calibration problems [62,63,64]. Both plasmonic (wavelength shifts due to target binding) and SERS (Raman fingerprints due to target binding) platforms will have to deal with batch consistency and pre-analytical variability issues before they can achieve their full potential [37,61]. In this context, detectability alone is not enough, because the readout also has to remain stable enough to support clinical interpretation.

For in vivo imaging, the modality needs to provide output information which can be used operationally. Quantitative CT scans, such as dual-energy CT and spectral CT scans, may be calibrated to produce voxel-level gold maps and validated by elemental quantification techniques such as ICP-MS analysis. Such quantitative CT images could then be used to estimate the total uptake of gold nanoparticles as well as their spatial distribution to ensure correct irradiation planning and follow-up [65,66]. Optical methods and photoacoustic methods have shown great potentials for imaging gold nanoagents because they have a high sensitivity for detecting gold. However, due to their limited penetration depth, they are most useful for superficial lesions and intraoperative navigation instead of whole-body quantification [67,68]. In cases where the single-modality image alone cannot provide enough information for treatment, multi-modal designs are needed. Combining the distribution mapping of gold nanoparticles with anatomical delineation will help to reduce the uncertainty when interpreting the margin of tumors [69,70].

Decisions on all platforms will require consideration of uptake magnitude, intratumoral coverage and time-window parameters: total uptake at the intended activation timepoint should be provided since trigger dependent benefit cannot be interpreted when exposure is too low and escalating energy delivery is not possible [71]. Spatial heterogeneity should be reported through coverage or uniformity measures to reveal unsensitized niches behind mean uptake numbers and therefore “cold spots” [71]. Kinetics parameters including time to peak retention and clearance slope might be useful to better define imaging and activation scheduling especially for fractionated treatments over multiple days during which intratumoral distribution could have changed [72]. Standardization efforts will need to be based on a common calibration strategy, region definition rules and whether quantitative maps represent tumor-wide coverage or vascular rich compartments which have proven to differ between cohorts and impacted quantification [70,73].

Redox-associated probes add value when their signals are co-registered with AuNP distribution and interpreted within a defined activation window together with uptake, coverage, and retention kinetics [51]. Under these conditions, ROS-linked imaging no longer serves only as an auxiliary contrast layer, but can verify whether AuNP activation has produced biologically meaningful oxidative conversion in the tumor region that was actually exposed [74]. Upon ROS-triggered aggregation, the photoacoustic signal and near-infrared absorption increase, aligning the imaging contrast with following intervention strategy [75]. In radiosensitization settings, ROS-responsive probes are most informative when they confirm that irradiation-induced oxidant signals arise in Au-positive regions and coincide with subsequent damage-associated endpoints, thereby strengthening the biological interpretation of image-guided activation [76]. Meanwhile, fluorescence tracking might help to determine irradiation time points, and infrared thermography could monitor local heating during photothermal procedure [77].

**Figure 3:**
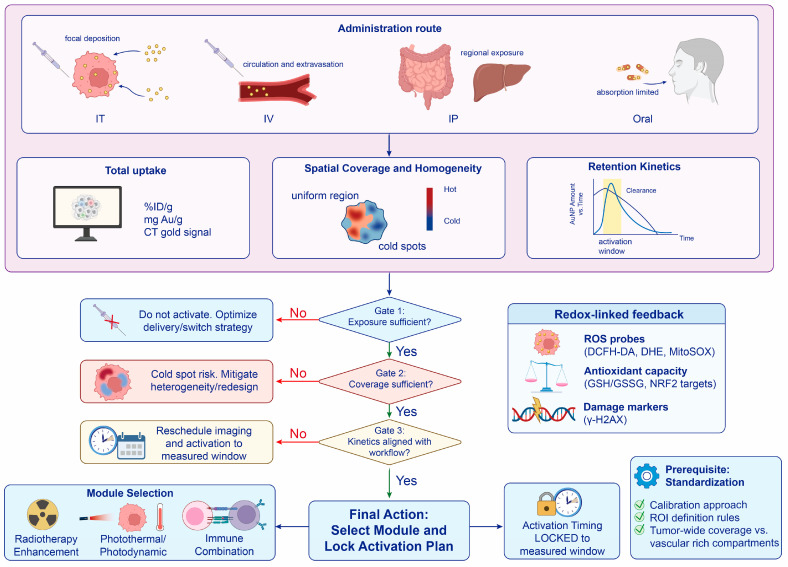
Quantification-guided workflow for AuNP activation. Tumor uptake, intratumoral coverage, and retention kinetics are used to decide whether and when an AuNP-based module should be activated. Activation is delayed when tumor exposure is insufficient, reconsidered when coverage is uneven, and timed to the interval in which tumor signal remains adequate while off-target signal remains acceptable. Redox-linked readouts then help verify whether activation has produced a measurable biological response.

Quantitative AuNP diagnostics translate uptake, coverage, and kinetics into stratification variables and define the feasible window for irradiation or energy-triggered activation, providing the prerequisites for interpreting and optimizing AuNP-enabled therapy (Figure 3).

## 5. Therapy

### 5.1. Prerequisites for Activation Dependent Therapy

**Figure 4:**
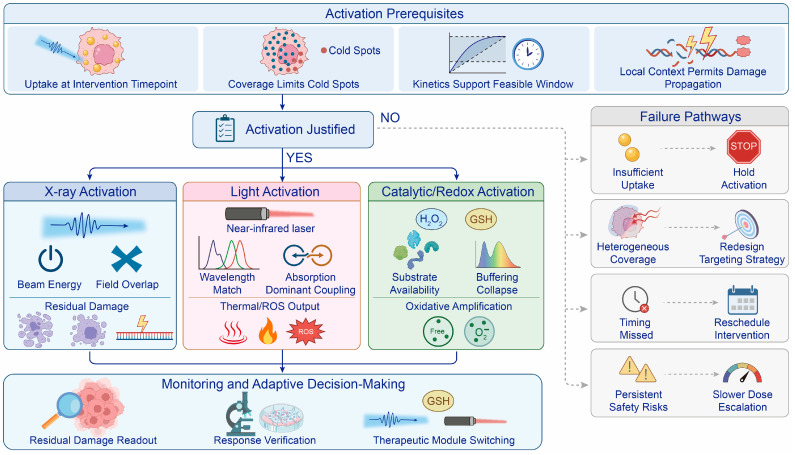
Risk–benefit map for activation-dependent AuNP therapy. AuNP activation is favored when tumor uptake, coverage, and retention align with the planned treatment window. Dose escalation or repeated activation requires closer monitoring when off-target accumulation or long-term persistence is evident. Poor uptake, dominant cold spots, or a missed activation window should prompt rescheduling or redesign instead of futile escalation.

AuNP-enabled therapy depends on whether particle deposition, activation, and tumor redox state are aligned within the same biologically relevant region. Sufficient uptake at the intended intervention timepoint is necessary, but it is not enough on its own. The particles must also cover the target volume, remain present during the activation window, and occupy compartments where the selected modality can generate an effective physical or chemical insult [78,79] (Figure 4). Under X-ray irradiation, this requires overlap between AuNP distribution, the radiation field, and damage-sensitive cellular targets. Under optical or catalytic activation, it further depends on light accessibility, substrate availability, and local antioxidant buffering. Therefore, mean tumor accumulation provides only limited guidance, because acceptable bulk uptake may still coexist with poor intratumoral coverage, activation-field mismatch, vesicular sequestration, hypoxia, or redox buffering that prevents transient ROS generation from becoming durable biological injury [8,32].

### 5.2. Activation Modules That Leverage Redox and Energy Conversion

Radiosensitization is one of the clearest settings in which AuNP-mediated redox amplification is initiated by a defined physical event. After photon absorption by gold, inner-shell ionization can trigger emission of short-range secondary electrons, including Auger electrons, which concentrate energy deposition within a narrow volume around each particle instead of distributing it evenly across the irradiated tissue [44,45]. That spatially confined energy release intensifies local water radiolysis and elevates reactive species formation only where the particle, the radiation track, and the biological target are brought into close proximity [45,46]. The mechanistic endpoint is not reactive species formation itself, but successful propagation of this nanoscale event into residual DNA damage, membrane oxidation, mitochondrial failure, or clonogenic loss within the Au-exposed tumor compartment. Radiosensitization therefore depends less on bulk Au uptake than on whether enough particles occupy the irradiated tumor volume and whether their subcellular localization places these electron cascades near compartments in which nanometric damage can be converted into persistent DNA injury or clonogenic loss [46,47].

**Figure 5:**
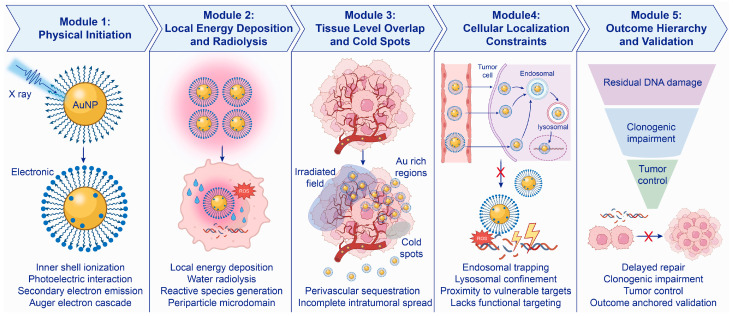
Radiosensitization framework linking AuNP localization to treatment outcome. AuNP-mediated radiosensitization begins with local energy deposition and ROS generation around particle sites, but treatment benefit depends on overlap with the irradiation field and vulnerable tumor regions. Particle density, beam energy, intratumoral cold spots, subcellular localization, and retention kinetics determine whether this local event progresses to residual DNA damage, clonogenic loss, tumor control, and, when relevant, immune activation.

At the tissue level, perivascular sequestration and incomplete intratumoral spread reduce the freely diffusible fraction of AuNPs and leave cold regions insufficiently sensitized, even when mean tumor uptake appears acceptable [80,81]. At the intracellular level, endosomal and lysosomal confinement means that higher cellular uptake does not automatically place physical initiation near DNA-relevant or mitochondria-relevant targets [82]. This has two practical implications for engineering. Firstly, formulations intended for broad tumor coverage should avoid excessive membrane affinity during the circulation and early extravasation stages, and should instead rely on staged or conditionally exposed penetration-promoting features after tumor entry. Secondly, uptake-promoting ligands should, where possible, be paired with escape-enabling or trafficking-directing elements, such as pH-responsive membrane-disruptive components, fusogenic motifs, photochemical internalization strategies, or organelle-directed modules, because uptake without productive intracellular access may still leave the radiosensitizing gain biologically underused. Accordingly, biological validation should establish that local physical amplification has been converted into persistent damage by demonstrating residual γ-H2AX or 53BP1 foci, delayed repair, and clonogenic impairment rather than particle presence alone [50]. In other words, the key question is whether particle localization can be carried forward into lasting biological injury. Some researchers also found that gold nanoparticle plus radiotherapy could produce some signals indicating immunogenic cell death. However, in order to show the benefit of systemic immunity, demonstration of systemic immune benefit requires evidence that these signals promote recruitment of mature dendritic cells or cytotoxic T cells into tumors. In addition, many tumors, such as gliomas, provide an immunosuppressive environment with physical barriers preventing immune cells entering the tumor [80] (Figure 5).

In practice, the most effective designs usually integrate three coordinated functions within the same tumor compartment [75,78]. One function is substrate reinforcement, for example by continuously supplying H_2_O_2_ or related reactants to prevent rapid exhaustion of the catalytic feedstock. A second function is redox buffering collapse, typically through GSH consumption or interference with GSH regeneration, because high glutathione levels in the tumor milieu can otherwise neutralize newly generated oxidants before durable injury develops [83,84]. A third function is spatial or microenvironmental gating, so that catalytic activity is preferentially turned on under tumor-associated conditions rather than expressed constitutively in circulation or normal tissues [85]. Catalytic systems gain stronger mechanistic support when ROS generation is linked to sustained substrate supply, reduced antioxidant buffering, and downstream cellular injury within the same tumor compartment. More persuasive evidence therefore comes from coordinated readouts that include ROS elevation, GSH depletion, mitochondrial depolarization, apoptosis or lipid damage, and eventual tumor suppression [21,75,78].

Photothermal and photodynamic modules are best interpreted as morphology-sensitive and compartment-sensitive redox interventions. Their performance depends on where the plasmon band lies relative to the excitation wavelength, how strongly the particle absorbs rather than scatters incident light, how uniformly the tumor is covered, and whether activation coincides with peak intratumoral retention. Shape is therefore a primary determinant of photothermal activation because aspect ratio, edge geometry, and branch architecture define resonance mode structure and near-field concentration. Size remains important even when resonance wavelength is held approximately constant, because it still alters light-to-heat conversion efficiency rather than simply shifting the optical band [26]. Surface chemistry further modulates photothermal performance by controlling colloidal stability in biological media, nanoparticle packing after cellular uptake, and subcellular confinement, all of which influence whether plasmonic heating remains focal and whether it is released in the tumor compartment that matters biologically [51,86]. In 3D tumor spheroids, nanoscale heating generated by photoexcited gold nanostars was associated with spatially heterogeneous microscale damage, supporting the view that photothermal efficacy cannot be inferred from laser settings or bulk uptake alone [49]. In plasmon-enhanced photodynamic therapy, AuNP-photosensitizer coupling is likewise geometry-dependent and distance-dependent, because structural arrangement determines whether near-field amplification increases ROS generation or whether thermal and structural perturbation weakens that coupling during sequential treatment [86]. These design variables can red-shift or damp the resonance band through structural and dielectric effects, which in turn alters optical coupling and the final photothermal output achieved in the tumor compartment [87,88].

### 5.3. Translation Constraints and Regimen Design

Engineering strategies are best aligned with the dominant loss step along the delivery-conversion cascade. Systems limited by premature clearance benefit from circulation-preserving design, because inadequate blood residence constrains tumor exposure regardless of ligand choice [86]. When the main loss arises from poor intratumoral transport, size-transformable, charge-convertible, matrix-modulating, or ligand-unmasking platforms can improve movement beyond perivascular regions and reduce peripheral sequestration [86]. When the major restriction appears after cellular uptake, greater value comes from endosomal escape and directed intracellular trafficking, since uptake alone often leaves a large fraction of particles confined within vesicular compartments and functionally inaccessible [82,89]. Under these conditions, stimuli-responsive platforms help the formulation meet sequential transport requirements across circulation, tumor penetration, cell entry, and intracellular access [80,90].

The safety and feasibility of these safety and feasibility considerations complete the translational evaluation of therapy. The off-target accumulation of AuNPs may cause organ damage because liver and spleen are the main filter organs, which can reduce the therapeutic window of AuNPs [52]. If there is a possibility of persistence and bioaccumulation of non-degradable gold materials, long-term monitoring should also be considered. When AuNPs are used in clinic, the diagnosis-to-therapy link will become direct. Quantitative uptake, coverage, and kinetics determine whether the AuNP-enabled module is suitable for use and when activation should happen, and also prevent misattribution of negative outcomes to biology when delivery prerequisites are missing [22,78].

Because efficacy depends on exposure, coverage, and time-window alignment, tracking delivery metrics together with redox-associated readouts during treatment can identify suboptimal response patterns early and support adaptive scheduling or module switching before failure becomes clinically overt [71,73].

## 6. Prognosis

Quantifiable delivery and response variables can be used as decision inputs instead of being descriptive outputs, which means AuNP-based theranostics can also serve as predictive and monitoring tools. Biomarkers can be divided into two types in oncology: one is prognostic biomarker, and the other is predictive biomarker. Prognostic biomarker can reflect the development of disease itself, regardless of treatment, while predictive biomarker could estimate the possibility of benefit from a certain therapy after intervention and enable patient stratification and personalized adjustment [10,91]. One example of the application of nanomedicine based on imaging-detectable companion nanoparticles before the administration of therapeutic nanoparticles has been reported. Delivery and retention of the diagnostic particles were used to predict the delivery and efficacy of the following therapeutic module, thus shifting stratification prior to treatment [92]. In this case, Au burden, spatial coverage, and kinetics can be used to judge whether activation is likely to produce meaningful benefit, including adequate tumor delivery, sufficiently uniform intratumoral distribution, and coverage within the effective time window [92]. However, it is unreliable to infer nanoparticle extravasation and retention based on single physiological surrogates, such as vascular density, perfusion, collagen content, or interstitial pressure. Instead, imaging-derived uptake magnitude, spatial distribution, and kinetic parameters can be used to judge whether to use a therapeutic nanoparticle module and whether another strategy should be added. Lesions with poor uptake may be unsuitable for nanoparticle-enabled therapy; if high but heterogeneous uptake occurs, regional hyperthermia can be used to improve homogeneity so as to avoid suboptimal efficacy caused by heterogeneous intratumoral distribution [91,93].

Monitoring during treatment is meant to detect signs of non-response at an early stage so as to avoid late failure. However, many cases of durable control failure occur after measurable changes in the exposure and spatial distribution have occurred; therefore, quantitative readouts should be used to assess risks instead of being regarded as descriptive imaging. Total tumor uptake at clinically relevant timepoints can serve as a crude indicator of whether a lesion is likely to behave as a low- or high-risk site for recurrence under standard care assumptions [71]. This stratification can be further refined by incorporating spatial heterogeneity. Coverage and uniformity metrics identify unsensitized niches that can persist as residual disease even when mean uptake appears adequate, and these niches can dominate relapse risk. However, heterogeneity is most informative when expressed as the fraction of tumor volume above a level associated with measurable tumor-damaging effects and linked to specific anatomic subregions that may be responsible for particular failure patterns [72]. In addition, time-to-peak and clearance slope could help define when measurements are most informative for distinguishing true lack of delivery from delayed delivery, and could be used to guide timing of follow-up imaging to allow detection of residual disease before clinical progression. Kinetics are important because without them, isolated snapshots can be misleading. The same uptake value could represent very different states of delivery and clearance [73]. In order for there to be cross-center comparability, it must be reported what type of calibration was used, what region-definition rules were applied, and whether maps capture tumor-wide coverage or only vascular-rich compartments, as these will affect uptake and heterogeneity estimates and therefore may lead to opposite risk classifications between cohorts [71,73].

After treatment, surveillance and early recurrence-risk management follow naturally from the same quantification logic and can be interpreted in a prevention-oriented way. A proactive secondary-prevention approach uses high-sensitivity imaging and biomarkers to identify microscopic residual disease and suboptimal responses at the earliest possible stage, then personalizes and adjusts management rather than relying on a single post-treatment evaluation [94]. In a quantifiable system, tracking intra-tumoral nanoparticle accumulation and spatial distribution immediately before or during therapy, together with non-invasive monitoring of temperature and thermal dose in photothermal settings, can be correlated with tissue necrosis and longer-term outcomes to define warning signs and prespecified intervention-triggering levels. These quantitative signals can then be translated into early-correction rules during or immediately after treatment. Uptake, coverage, kinetics, and redox-associated monitoring signals therefore function as practical triggers for intensified surveillance and timely intervention against residual disease [94,95].

## 7. Prevention

The first is early screening and risk monitoring in high-risk settings, where the primary value is not a one-time sensitive test but a low-burden, repeatable measurement that remains quantitative and comparable across time. In this context, AuNP-enabled readouts should be understood as quantitative follow-up signals rather than descriptive images. Consequently, uptake, coverage, and kinetics can be used to determine feasibility, schedule follow-up assessments, and define trigger rules for escalation or de-escalation of AuNP-enabled modules [71,73].

The second scenario is secondary prevention after surgery or after an apparent response, where the objective is to prevent recurrence and metastasis through early detection of microscopic residual disease and timely course correction. A proactive approach is described as using high-sensitivity imaging and biomarkers to identify suboptimal responses at the earliest possible stage and then personalize and adjust management rather than relying on a single post-treatment evaluation [94]. In a quantifiable system, intra-tumoral nanoparticle accumulation and spatial distribution can be tracked immediately before or during therapy, and, for energy-based interventions, non-invasive monitoring of temperature and thermal dose can be integrated. Correlating these quantitative signals with histological necrosis and longer-term outcomes helps define intervention-triggering levels and generates actionable triggers for immediate correction, such as performing an additional ablation in a region that did not reach the desired temperature or giving an extra radiation boost to an area with low nanoparticle uptake before the patient leaves the treatment setting [95]. At that stage, prevention is no longer abstract follow-up, but early correction guided by measurable residual risk.

Oxidative stress should remain a secondary but useful layer in prevention because it can serve as both a trigger and a safety-relevant endpoint. This rationale is reflected in a postoperative hydrogel system that combines photothermal therapy with ROS-scavenging and VEGF capture, so that local cytoreduction is paired with control of oxidative and pro-angiogenic cues in the surgical bed [86].

Prevention of long-term injury requires minimization of off-target toxicity, as this will minimize late effects following primary treatment. That is, the sensitizing effect should be mainly located at the tumor site by targeted drug delivery and controlled drug release, so as to decrease the exposure of normal tissues. When the targeting efficiency or biodistribution is poor, high-dose inorganic nanoparticles will accumulate in normal tissues and thus increase the possibility of side effects under some circumstances [96]. In addition, a more mature risk–benefit assessment has also highlighted long-term retention and chronic uncertainty as translational obstacles. Accumulation within the liver, spleen, and kidneys, and possible induction of chronic oxidative stress and inflammation are the main concerns; therefore, prolonged follow-up at regular intervals is warranted, e.g., liver and kidney function testing. This emphasizes the need for minimal effective dosing and design that promotes degradation or clearance wherever possible [97,98]. All of the proposed prevention pathways converge around a clinical translation checklist, rather than relying on other mechanisms. Prevention-oriented protocols require standardized quantification that is repeatable over time, explicit trigger rules that link quantitative readouts to intensified surveillance or low-intensity corrective interventions, and feasibility constraints that account for manufacturing complexity, batch consistency, and cost. Intensive monitoring and personalized adjustment need to be justified in terms of expected clinical benefit; otherwise, even sound preventive approaches will not find their way into routine oncology workflows [98,99].

## 8. Future Perspectives and Challenges

Predictability at the patient level remains the key challenge for AuNP-oxidative-stress theranostic development, and the major limiter is delivery. Variations in tumor accumulation and intratumoral penetration among patients and tumors may cause failure of such a theranostic platform due to insufficient or uneven exposure and coverage despite promising preclinical data. Therefore, deployability should be regarded as a prerequisite of theranostic devices, and their activation should be determined according to the quantified uptake, spatial distribution and kinetics of these particles, instead of their formulations alone.

However, reproducibility is also challenged by what happens after injection. Protein corona formation reshapes NP biological identity, alters immune recognition and clearance, and can mask targeting ligands; therefore, in vitro behavior may be hard to translate into in vivo behavior. Further progress will depend on corona-aware characterizations that are both standardized across labs and linked to measurable exposure and outcome variables, so that results remain comparable across batches and center.

Oxidative stress will add clinical value only if it becomes quantitative and decision-relevant in contrast to being used merely as a post hoc explanatory variable. Early-stage redox PET work demonstrates the feasibility of non-invasive oxidative stress imaging for monitoring and personalization, but there is still a need to determine which minimum set of redox linked response readouts, with calibration of their timing and action thresholds, are required to verify whether AuNP activation resulted in the intended redox shift at places where AuNP actually accumulated. Regarding radiosensitization, there is still uncertainty regarding multiscale matching because reactive species formation is very localized and macroscopic effect depends on microscopic distribution and field overlap which reinforces the need to combine models with measurement of microdistribution and durable endpoint.

## 9. Conclusions

Gold nanoparticle theranostics should not be understood as a simple combination of imaging capability and therapeutic potential. Their real value lies in how nanoparticle delivery and tumor redox biology interact across the entire disease course. This review has shown that oxidative stress in tumors is not a fixed background state, but a dynamic system shaped by ROS production, antioxidant buffering, spatial compartmentalization, and damage thresholds within the tumor microenvironment. Against this background, the significance of AuNPs depends not only on their physical or catalytic properties, but also on whether they can reach the tumor in sufficient amount, distribute across clinically relevant regions, persist through the intended intervention window, and localize in cellular contexts where activation can be converted into meaningful biological effects.

Seen from this perspective, diagnosis, therapy, prognosis, and prevention are not separate applications, but different clinical expressions of the same operational logic. In diagnosis, quantitative imaging and redox-associated readouts are needed to determine whether an AuNP-enabled intervention is feasible and when activation should occur. In therapy, benefit depends on whether exposure, spatial coverage, and activation timing are properly aligned, and whether local physical or chemical amplification is actually translated into persistent damage rather than transient signal change. In prognosis and prevention, the same variables become tools for risk stratification, response monitoring, early correction, and surveillance of residual or recurrent disease. The central message of this review is therefore that AuNP theranostics should be interpreted through measurable delivery and response variables rather than through formulation design or total tumor accumulation alone.

Mechanistically, AuNP-enabled therapy should be interpreted as a delivery-to-damage sequence rather than as a formulation property alone. AuNP localization defines tumor exposure and retention at the planned intervention time, the activation modality determines the initiating physical or catalytic event, and the local redox threshold determines whether this initiation propagates into durable injury. Practical protocols should therefore be based on measurable exposure and response checkpoints, including tumor Au burden, spatial coverage, cold-spot burden, retention kinetics, redox-response signals such as ROS, lipid peroxidation or GSH/GSSG changes, damage endpoints such as persistent γ-H2AX or 53BP1 foci and clonogenic loss, and normal-organ accumulation.

The path toward clinical use will depend on how successfully this framework can be standardized and validated in real patients. Future progress requires more than stronger ROS amplification or more complex nanoparticle design. A more important task is to determine when delivered gold is actually positioned to produce useful biological effects. This requires reproducible quantification of tumor uptake, spatial coverage, retention, and intracellular accessibility, together with redox-linked readouts that show whether activation has exceeded local antioxidant buffering and produced persistent damage rather than a transient ROS signal. Clearer criteria for activation, better control of batch consistency and in vivo behavior after administration, and more credible long-term safety assessment will be essential before AuNP platforms can be used to guide treatment decisions with confidence. AuNP platforms will have the greatest clinical impact when they can do more than generate signal or enhance local damage in experimental models. They must support treatment selection, guide activation at the right time, explain response or non-response with confidence, and ultimately improve patient management across diagnosis, intervention, follow-up, and recurrence prevention.

## Figures and Tables

**Table 1:** Operational quantitative variables for threshold assessment and decision making in AuNP-enabled theranostics.

Term	Definition	Representative Readouts
Threshold crossing	Effective tumor injury boundary	exposure + redox + damage
Tumor uptake	Au burden at activation	%ID/g, mg Au/g, CT gold signal
Coverage/heterogeneity	Spatial distribution adequacy	coverage fraction, uniformity, cold spots
Retention kinetics	Delivery–activation alignment	time to peak, retention, clearance slope
Redox-linked response	Oxidative activation readout	ROS signal, lipid peroxidation, oxidative DNA damage, GSH/GSSG
Damage	Biological damage endpoint	residual γ-H2AX, 53BP1, apoptosis, clonogenic loss
Safety indicators	Normal-tissue risk readout	organ uptake, blood toxicity, organ toxicity, persistence

**Table 2:** Route-dependent constraints on AuNP exposure and tumor localization.

Route	Circulation/Residence	RES or MPS Uptake	Extravasation	Tumor Exposure	Main Limitation
Intravenous	Requires prolonged circulation	High	Required	Variable and often heterogeneous	Liver and spleen sequestration, uneven penetration [27,28]
Intratumoral	Minimal circulation requirement	Limited initially	Not required for initial localization	High local but focal	Incomplete spread and leakage [28,29]
Oral	Absorption-dependent	Often redistributed after absorption	Usually still required for extra-GI tumors	Low to variable	GI degradation and poor absorption [27]
Intraperitoneal	Regional residence is more important than blood circulation	Peritoneal macrophage uptake is relevant	Lower for peritoneal nodules	High regional exposure in peritoneal disease	Cavity clearance and uneven nodule coverage [30,31]

## Data Availability

No new data were created or analyzed in this study. Data sharing is not applicable to this article.

## Abbreviations

Abbreviation	Full term
AuNPs	gold nanoparticles
ROS	reactive oxygen species
GSH	glutathione
GSSG	oxidized glutathione
GPx	glutathione peroxidase
GPXs	glutathione peroxidases
SOD	superoxide dismutase
NADH	nicotinamide adenine dinucleotide
NADPH	nicotinamide adenine dinucleotide phosphate
NOX	NADPH oxidase
KEAP1	Kelch-like ECH-associated protein 1
NRF2	nuclear factor erythroid 2-related factor 2
ARE	antioxidant response element
Trx	thioredoxin
TrxR	thioredoxin reductase
Prx	peroxiredoxin
Srx	sulfiredoxin
CAFs	cancer-associated fibroblasts
MDSCs	myeloid-derived suppressor cells
TAMs	tumor-associated macrophages
Treg	regulatory T cell
SERS	surface-enhanced Raman scattering
CT	computed tomography
ICP-MS	inductively coupled plasma mass spectrometry
PDT	photodynamic therapy
PTT	photothermal therapy
Ce6	chlorin e6
ICD	immunogenic cell death
ER	endoplasmic reticulum
